# Vaginal estrogen therapy for treatment of menopausal genitourinary syndrome among breast cancer survivors: a systematic review and meta-analysis

**DOI:** 10.61622/rbgo/2025rbgo46

**Published:** 2025-07-15

**Authors:** Gabriel Moreira Lima Santos, Augusto Ostermann Magalhães, Pedro do Valle Teichmann, Maria Celeste Osório Wender

**Affiliations:** 1 Hospital de Clínicas de Porto Alegre Serviço de Ginecologia e Obstetrícia Porto Alegre RS Brazil Serviço de Ginecologia e Obstetrícia, Hospital de Clínicas de Porto Alegre, Porto Alegre, RS, Brazil.; 2 Universidade Federal do Rio Grande do Sul Departamento de Ginecologia e Obstetrícia Porto Alegre RS Brazil Departamento de Ginecologia e Obstetrícia, Universidade Federal do Rio Grande do Sul, Porto Alegre, RS, Brazil.

**Keywords:** Estrogen replacement therapy, Estrogens, Hormone replacement therapy, Menopause, Genitourinary syndrome, Cancer survivors, Breast neoplasms

## Abstract

**Objective:**

Assess survival outcomes and risk of recurrence in vaginal estrogen therapy (VET) users with medical history of breast cancer.

**Data source:**

The search strategy was guided by standardized terms and keywords were identified from controlled vocabularies. Following databases were used for literature search: Pubmed, EMBASE, Cochrane, Scopus and Web of Science. Only studies published in the 21st century (2001–present) and written in English were included.

**Study selection:**

A total of 988 records were reviewed by two independent authors. After full-text analysis of 38 of them, 7 articles were included in the meta-analysis.

**Data collection:**

Data from eligible studies were extracted and tabulated based on predefined criteria: author, country, year, study type, sample size, type of intervention, use of aromatase inhibitors, duration of follow-up, and main outcomes.

**Results:**

118.659 breast cancer survivors were analyzed, of whom 6.358 were treated with VET. The overall analysis showed no significant increase in the risk of recurrence (RR = 0.87, 95%CI: 0.67-1.11). VET users had a significant reduction in all-cause mortality (RR = 0.80, IC95%: 0.75–0,86).

**Conclusion:**

Vaginal estrogen therapy appears to be safe in the management of menopausal genitourinary syndrome in breast cancer survivors and it is related to significantly lower all-cause mortality.

**Prospective Register of Systematic Reviews (PROSPERO):**

CRD42024602047

## Introduction

Breast cancer is the most incident tumor among women and it is the leading cause of cancer-related death.^(1)^ Due to advances in screening, diagnosis and treatment there was a significant increase in survivorship of the patients, however, it still impacts their overall quality of life (QoL) and addresses a potential health issue.^(2)^

One of the main conditions that may impact on breast cancer survivor's QoL is the Genitourinary Syndrome of Menopause (GSM), a disorder related to estrogen deficiency, which encompasses vulvovaginal and urinary symptoms, including dryness, burning sensation, dyspareunia, urinary urgency and recurrent infections.^(3)^ Studies indicate that women with a history of breast cancer have a 20% higher incidence of GSM compared to postmenopausal women without cancer.^(4)^

The symptomatology of GMS is progressive if untreated and is exacerbated in patients undergoing therapies such as AIs, commonly used as adjuvant hormonal treatment in postmenopausal women.^(4)^

Estrogen-based local therapy is considered first-line treatment in postmenopausal patients without history of breast cancer, providing a good relief of the symptoms, preventing progression of the disease and improving the QoL.^(5)^ In breast cancer survivors, it still remains a concern due to the hypothetical impact on risk of recurrence.^(6)^ Recent studies, however, suggest that low-dose VET does not significantly increase systemic estradiol levels, keeping them below 30 pg/mL, typical of the postmenopausal period.^(7)^

According to the American College of Obstetricians and Gynecologists (ACOG), the initial management of symptoms in patients with a history of breast cancer should prioritize non-hormonal therapies, such as silicone-based lubricants, vaginal moisturizers with hyaluronic acid or polycarbophil, and vitamin E and D suppositories. Although effective, these methods do not always provide complete relief.^(5)^

Although VET's effectiveness in relieving GSM symptoms is well established, there is a lack of long-term data on its use in women with a history of breast cancer. This systematic review aims to evaluate the safety of topical estrogen use for vaginal atrophy in this population, focusing on overall survival and recurrence-free survival outcomes.

## Methods

A systematic review and meta-analysis were conducted to evaluate the safety of vaginal estrogen therapy in treating genitourinary syndrome in patients with a history of breast cancer. The protocol was developed following the Preferred Reporting Items for Systematic Reviews and Meta-Analysis Protocols (PRISMA-P) and was registered in the International Prospective Register of Systematic Reviews (PROSPERO) under the number CRD42024602047. The search strategy was guided by standardized terms and the acronym framework, and keywords were identified from controlled vocabularies: Health Sciences Descriptors (DECS) via the Virtual Health Library Regional Portal, Medical Subject Headings (MESH) through PubMed, and Emtree (Embase subject headings) from the EMBASE (Elsevier) database. A preliminary search was performed to identify additional terms in the titles, abstracts, and keywords of the articles, followed by the application of a definitive search strategy across all databases.

The search strategy was applied to the following databases: PubMed/MEDLINE, EMBASE, Cochrane, Scopus, and Web of Science. The following search terms were used: (vaginal estrogen OR estradiol OR estriol OR promestriene) AND (atrophic vaginitis OR vaginal atrophy OR vulvovaginal atrophy OR genitourinary syndrome OR urogenital atrophy) AND (breast cancer). Detailed search strategies for each platform are included in Supplementary Material (Table 1S)

Ethical approval was not required as this review was based on publicly available scientific literature. Traditional dissemination strategies were employed, including open-access peer-reviewed publications, scientific presentations, and reports.

Observational studies that described the risk of recurrence and/or mortality associated with vaginal estrogen therapy for genitourinary syndrome in patients following breast cancer treatment were included. Only studies published in the 21st century (2001–present) and written in English were included. Case reports, abstracts, review articles, commentaries, and letters to the editor were excluded.

**Figure 1:**
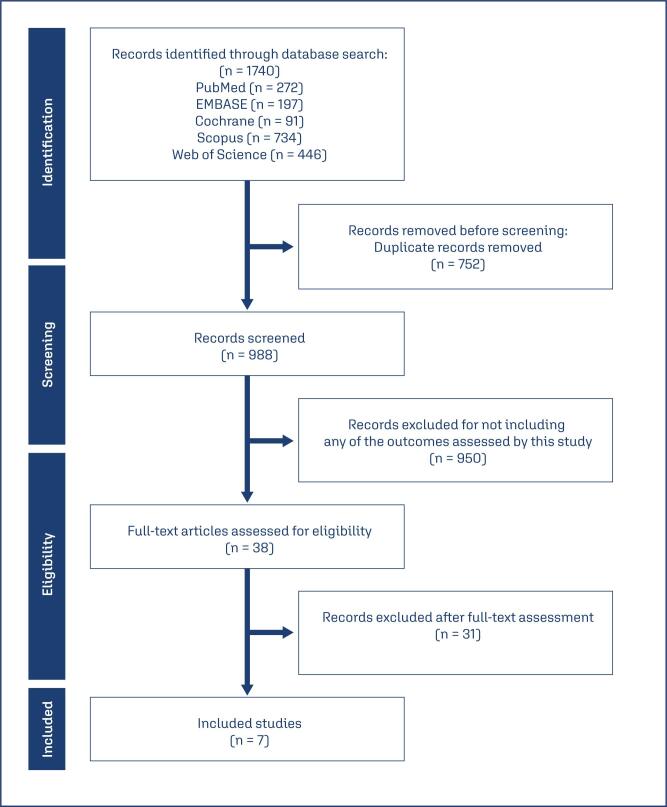
PRISMA fluxogram for literature search and study selection

All selected articles were exported to the Intelligent Systematic Review software (Rayyan) for selection based on the inclusion criteria. After removing duplicates, the titles and abstracts were independently screened by two reviewers (GMLS and PVT), who assessed the selected texts for compliance with the inclusion criteria. The full texts of these potentially eligible studies were independently retrieved and evaluated for eligibility by two review team members (GMLS and PVT). Only studies identified by both reviewers as meeting the inclusion criteria were ultimately included in the systematic review, and a third reviewer (MCOW) made the final inclusion decision in case of discrepancies. In results of the study selection and exclusion process were summarized using the PRISMA flowchart, as shown in figure 1.

Data from eligible studies were extracted and tabulated based on predefined criteria: author, country, year, study type, sample size, type of intervention, use of aromatase inhibitors, duration of follow-up, and main outcomes. The Newcastle-Ottawa Scale (NOS) was used to assess the quality of the included studies across three domains: selection, comparability, and outcomes. Studies scoring 6–9 points were considered as high-quality data, while those scoring 3–5 were deemed moderate quality. A funnel plot was used to evaluate the risk of publication bias, and Egger's test was applied to assess funnel plot asymmetry. In this article, the included papers received scores between 7 and 8, indicating high quality. The detailed resume of NOS assessment can be encountered in Supplementary Material (Table 2S).

Meta-analyses were conducted using a random-effects model with the inverse variance method to calculate risk ratios (RRs) and 95% confidence intervals (CI) via the platform available at [https://metaanalysisonline.com]. Heterogeneity was assessed using the I^2^ statistic and chi-square test (p &lt; 0.05). An I^2^ value between 50–90% indicated substantial heterogeneity. Sensitivity analyses were performed to explore potential sources of heterogeneity.

## Results

A total of 1,740 articles were identified through the searching strategy. After removing duplicate entries (n = 752) and articles whose titles/abstracts did not meet our PICOT criteria (n = 950), we fully reviewed all potentially relevant studies (n = 38). Of these, 31 studies were excluded due to the absence of relevant outcomes (Figure 1). At the end, seven studies were included in this meta-analysis, encompassing 118,659 women with a history of breast cancer, of whom 6,358 received vaginal estrogen therapy for the treatment of genitourinary syndrome of menopause (GSM). The main characteristics and results of these studies are summarized in Table 1. Notably, three of the included studies involved patients who were also using aromatase inhibitors (AIs), enabling a specific subgroup analysis regarding this concomitant treatment.

**Table 1:** Baseline characteristics of included studies

Autor, year and country	Type of study	Sample size (n)	Intervention	Patients receiving intervention (n)	Aromatase inhibitor use	Mean follow up (years)	Quality assessment (NOS)	Conclusion
O’Meara et al. (2001)^(8)^ USA	Cohort	2775	Conjugated Equine Estrogens and Dienestrol	75	NA	4-5	8	It was observed that HRT after breast cancer has no adverse impact on recurrence and mortality.
Durna et al. (2002)^(9)^ Australia	Cohort	1122	Estriol vaginal cream 0.5mg and Estradiol cream 25mcg	32	NA	6	7	HRT use for menopausal symptoms by women treated for invasive breast cancer is not associated with an increased risk of breast cancer recurrence or shortened life expectancy
Dew et al. (2003)^(10)^ Australia	Cohort	1472	Estriol cream and vaginal pessary and Estradiol cream 25mcg	69	NA	5,5	7	Topical estrogen usage does not appear to be associated with an increased risk of recurrence of breast cancer
Le Ray et al. (2012)^(11)^ United Kingdom	Case-control	13479	NA	271	NA	2-3	4	The use of LHT is not associated with an increase in breast cancer recurrence among women receiving a hormone therapy
Cold et al. (2022)^(12)^ Denmark	Cohort	8461	NA	1249	Yes	9-15	8	In postmenopausal women diagnosed with early stage hormonal receptor positive BC, neither VET nor MHT was associated with increased risk of recurrence or mortality. A subgroup analysis revealed an increased risk of recurrence, but not mortality in patients using AI.
Agrawal et al. (2023)^(13)^ USA	Cohort	42113	Conjugated Equine Estrogens and Estradiol Vaginal Cream	2111	Yes	5-10	8	There was no increased risk of breast cancer recurrence within 5 years in women with a personal history of breast cancer who were using vaginal estrogen for genitourinary syndrome of menopause
McVicker et al. (2024)^(14)^ Scotland and Wales	Cohort	49237	Estradiol vaginal cream 25mcg	2551	Yes	5-8	8	No evidence of increased early breast cancer-specific mortality in patients who used vaginal estrogen therapy compared with patients who did not use HRT was observed.

NA: not applicable; HRT: Hormone replacement therapy; LHT: Local hormonal therapy

**Figure 2:**
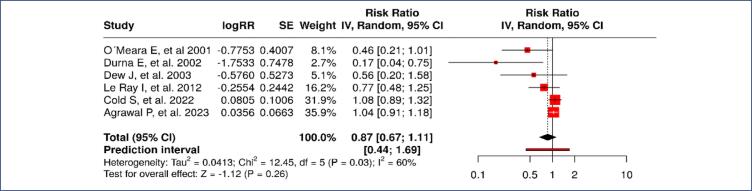
Risk of recurrence of breast cancer in patients who used VET

The overall pooled analysis of breast cancer recurrence in patients who used VET revealed no statistically significant increase in the risk of recurrence. As shown in figure 2, the pooled risk ratio (RR) was 0.87 (95% confidence interval [CI]: 0.67–1.11), with significant heterogeneity detected between studies (I^2^ = 60 %, p = 0.03). This heterogeneity suggests that variations in study populations and protocols contributed to the divergent results between studies. These findings support the safety of VET in patients with a history of breast cancer without concomitant use of aromatase inhibitors (AI).

**Figure 3:**
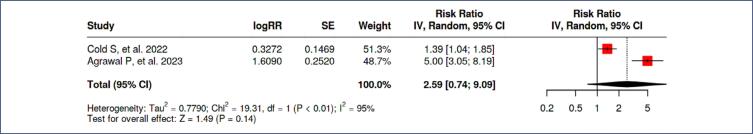
Risk of recurrence of breast cancer in patients who used VET and AI concomitantly

In the subgroup analysis of patients who used IA concomitantly with VET (Figure 3), no statistically significant increase in breast cancer recurrence risk was observed (RR = 2.59, 95% CI: 0.74–9.09).

**Figure 4:**
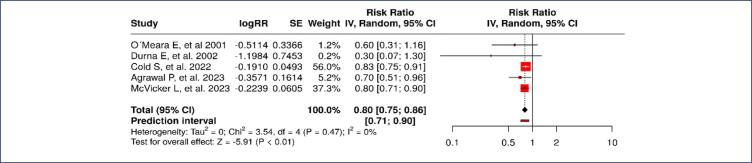
All-cause mortality in patients with a history of breast cancer who used vaginal estrogen therapy

For all-cause mortality, pooled analysis of the general population of breast cancer survivors who used VET showed a significant risk reduction of all cause mortality. As shown in figure 4, the overall risk ratio (RR) was 0.80 (95% CI: 0.75–0.86, p &lt; 0.05), without significant heterogeneity (I^2^ = 0%). This consistent effect across studies suggests that the use of topical vaginal estrogen may have a protective effect on overall survival in this group of women, possibly due to its role in improving quality of life and symptom management.

**Figure 5:**
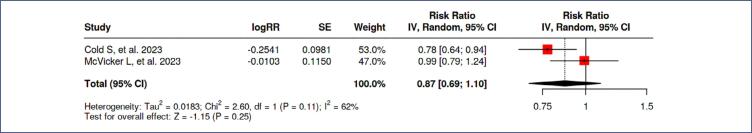
Mortality in breast cancer survivors who used VET associated with Ais

In contrast, the subgroup analysis of patients who used AIs along with VET, as shown in figure 5, did not demonstrate a statistically significant difference in all-cause mortality. The pooled risk ratio (RR) was 0.87 (95% CI: 0.69–1.10), with no significant heterogeneity detected (I^2^ = 0%). This suggests that, in this subgroup, vaginal estrogen therapy does not significantly influence overall mortality rates, and its use can be considered safe from a mortality perspective.

**Figure 6:**
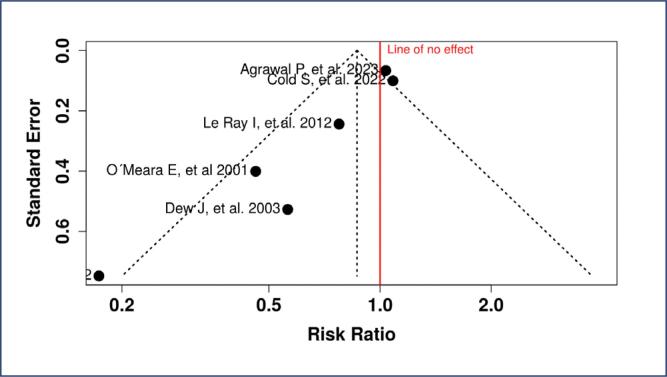
Funnel Plot A

Publication bias was assessed using funnel plots and Egger's test. Funnel Plot A (Figure 6) corresponds to the analysis of breast cancer recurrence. The slight asymmetry observed in the plot suggests potential publication bias, as smaller studies with non- significant results may have been underreported. This bias was confirmed by Egger's test

(intercept: −2.04, 95% CI: −2.85 to −1.23, t = −4.952, p = 0.008).

**Figure 7:**
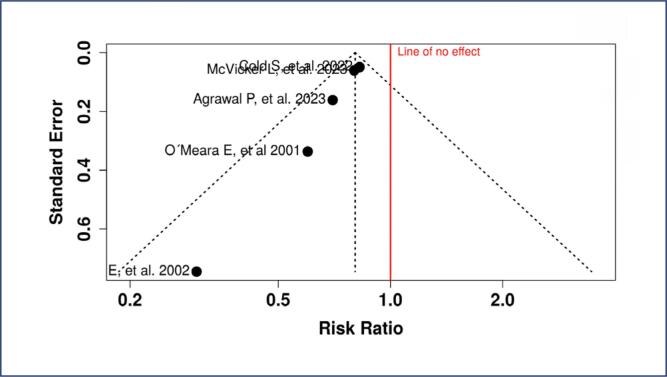
Funnel Plot B

Funnel Plot B (Figure 7) illustrates the analysis of all-cause mortality and reveals evidence of asymmetry, indicating potential publication bias. This was further supported by Egger's test, which detected significant asymmetry (intercept: −1.33, 95% CI: −1.6 to −1.06, t = −9.705, p = 0.002).

## Discussion

Vaginal estrogen therapy in breast cancer survivors has been a topic of interest due to concerns about the potential risk of cancer recurrence and impact on overall survival. This study analyzed large patient cohorts and found no evidence of an increased risk of breast cancer recurrence among VET users compared to non-users of hormonal therapy.

Additionally, it demonstrated a significant reduction in all-cause mortality in this population. Several clinical trials conducted in recent years have aimed to evaluate potential alterations in serum estrogen levels following the initiation of VET in women with a personal history of hormonal receptor-positive breast cancer, both in the absence of adjuvant AIs^(15)^ and during concurrent use of AIs with VET.^(16–20)^ These studies observed a transient increase in serum estradiol levels during the initial weeks of use; however, the levels remained insignificant and well below the postmenopausal threshold (≤ 30 pg/mL) when using low or ultra-low doses of estrogen-based vaginal products.^(4,7)^ These findings align with the results of this meta-analysis, which indicate no increased breast cancer recurrence risk among VET users compared to non-users of hormonal therapy.

A notable counterpoint arises from two cohort studies included in this review, which reported an increased recurrence risk associated with VET use in conjunction with AIs.^(12,13)^ In this subgroup, the pooled analysis of these two studies yielded a recurrence RR of 2.59 (95% CI: 0.74–9.09), but this result was not statistically significant and the analysis exhibited substantial heterogeneity (I^2^ = 95%). This high degree of heterogeneity implies that variations among studies, such as differences in AI protocols or patient selection criteria, may have contributed to the observed variance in recurrence risk. Additional well-designed studies are needed to better assess this risk in this specific subgroup of patients.

It is plausible that various factors are associated with the increased risk observed among AI users, such as the dose of vaginal estrogen, the dosage of aromatase inhibitors, or even the temporal relationship between the initiation of VET and the breast cancer diagnosis.^(13)^

Crandall et al.^(21)^ conducted an analysis within the Women's Health Initiative Observational Study and no increase in all-cause mortality risk was observed among vaginal estrogen users compared to non-users. In our pooled analysis of the general population with a history of breast cancer using VET, a statistically significant reduction in mortality risk was identified. We cannot directly infer a reduction in mortality, as this would require a more in-depth analysis of individual study characteristics and population variables.

These findings suggest that VET, which has minimal systemic absorption, may be considered a safe option for managing genitourinary symptoms of menopause in patients with a medical history of breast cancer without increasing overall mortality and recurrence.

However, it is important to note that long-term safety has not been fully established, particularly for patients on aromatase inhibitors. Individual clinical contexts should always be taken into account, and treatment options should be discussed based on each patient's specific characteristics.

This study presents several strengths, including a robust sample size and extended follow-up duration of the studied population. However, certain limitations must be highlighted, particularly regarding the interpretation of the results. Among these, potentially the most significant is the assessment of adherence to vaginal hormone therapy and its duration of use, variables that are often poorly measured or unavailable in studies, thus representing a potential source of bias.

Additionally, the patient profiles included in the analysis and the lack of adjusted analysis by cancer stage and histological grade in most included studies should be underscored. Furthermore, the absence of a control group for comparison purposes constitutes another limitation. It is also worth noting that in the included cohorts, patients with locally advanced or metastatic breast cancer were not considered candidates for local hormone therapy and were therefore excluded from the analysis. This exclusion may represent a potential bias, as the included patients, having less advanced disease, were already at lower mortality risk and consequently had longer survival.

## Conclusion

Vaginal estrogen therapy appears to be safe in the management of Genitourinary Syndrome of Menopause among breast cancer survivors, without impacting on recurrence risk and overall survival. Although all users concomitantly with VET must have a cautious approach due to the possible increase in the risk of recurrence. These findings highlight the importance of a shared decision between doctors and patients, especially in women taking aromatase inhibitors. Further research is necessary to assess long term outcomes and ensure the use of VET in breast cancer patients.

## Supplementary Material

**Table 1S:** Detailed searching strategy at each platform

Pubmed n=272 results	(Breast Neoplasms[mh] OR Breast Neoplasm*[tiab] OR Breast Tumor*[tiab] OR Breast Cancer*[tiab] OR Breast Carcinoma*[tiab] OR Cancer of Breast[tiab] OR Cancer of the Breast[tiab] OR Malignant Neoplasm of Breast[tiab] OR Breast Malignant Neoplasm*[tiab] OR Malignant Tumor of Breast[tiab] OR Breast Malignant Tumor*[tiab] OR Mammary Cancer*[tiab] OR Mammary Neoplasm*[tiab] OR Mammary Carcinoma*[tiab]) AND (((Atrophy[mh] OR Atroph*[tiab]) AND (Vagina[mh] OR Vagin*[tiab] OR Vulva[mh] OR Vulva*[tiab] OR Vaginal Diseases[mh] OR Vulvar Diseases[mh] OR Female Urogenital Diseases[mh] OR urogenital[tiab] OR vulvovagin*[tiab])) OR Atrophic Vaginitis[mh] OR genitourinary syndrome[tiab]) AND (Estrogens[mh] OR Estrogen*[tiab] OR oestrogen*[tiab] OR Estradiol[mh] OR Estradiol[tiab] OR Estriol[mh] OR Estriol[tiab] OR promestriene[nm] OR promestriene[tiab]) AND 2001:2024[dp] AND eng[la]
Embase n= 197 results	(‘breast tumor’/exp OR (‘Breast Neoplasm*’ OR ‘Breast Tumor*’ OR ‘Breast Cancer*’ OR ‘Breast Carcinoma*’ OR ‘Cancer of Breast’ OR ‘Cancer of the Breast’ OR ‘Malignant Neoplasm of Breast’ OR ‘Breast Malignant Neoplasm*’ OR ‘Malignant Tumor of Breast’ OR ‘Breast Malignant Tumor*’ OR ‘Mammary Cancer*’ OR ‘Mammary Neoplasm*’ OR ‘Mammary Carcinoma*’):ti,ab,kw) AND (((‘atrophy’/exp OR Atroph*:ti,ab,kw) AND ((‘vagina’ OR ‘vulva’ OR ‘vagina disease’ OR ‘vulva disease’ OR ‘urogenital tract disease’)/exp OR (Vagin* OR Vulva* OR urogenital OR vulvovagin*):ti,ab,kw)) OR ‘atrophic vaginitis’/exp OR ‘genitourinary syndrome’:ti,ab,kw) AND ((‘estrogen’ OR ‘estradiol’ OR ‘estriol’ OR ‘promestriene’)/exp OR (Estrogen* OR oestrogen* OR Estradiol OR Estriol OR promestriene):ti,ab,kw) AND [2001-2024]/py AND [english]/lim AND [embase]/lim NOT ([embase]/lim AND [medline]/lim)
Cochrane n = 91 results	([mh "Breast Neoplasms"] OR ((Breast NEXT Neoplasm*) OR (Breast NEXT Tumor*) OR (Breast NEXT Cancer*) OR (Breast NEXT Carcinoma*) OR "Cancer of Breast" OR "Cancer of the Breast" OR "Malignant Neoplasm of Breast" OR ("Breast Malignant" NEXT Neoplasm*) OR "Malignant Tumor of Breast" OR ("Breast Malignant" NEXT Tumor*) OR (Mammary NEXT Cancer*) OR (Mammary NEXT Neoplasm*) OR (Mammary NEXT Carcinoma*)):ti,ab,kw) AND ((([mh Atrophy] OR Atroph*:ti,ab,kw) AND ([mh Vagina] OR [mh Vulva] OR [mh "Vaginal Diseases"] OR [mh "Vulvar Diseases"] OR [mh "Female Urogenital Diseases"] OR (Vagin* OR Vulva* OR urogenital OR vulvovagin*):ti,ab,kw)) OR [mh "Atrophic Vaginitis"] OR genitourinary syndrome:ti,ab,kw) AND ([mh Estrogens] OR [mh Estradiol] OR [mh Estriol] OR (Estrogen* OR oestrogen* OR Estradiol OR Estriol OR promestriene):ti,ab,kw)
Scopus n = 734 results	TITLE-ABS-KEY("Breast Neoplasm*" OR "Breast Tumor*" OR "Breast Cancer*" OR "Breast Carcinoma*" OR "Cancer of Breast" OR "Cancer of the Breast" OR "Malignant Neoplasm of Breast" OR "Breast Malignant Neoplasm*" OR "Malignant Tumor of Breast" OR "Breast Malignant Tumor*" OR "Mammary Cancer*" OR "Mammary Neoplasm*" OR "Mammary Carcinoma*") AND TITLE-ABS-KEY((Atroph* AND (Vagin* OR Vulva* OR urogenital OR vulvovagin*)) OR "genitourinary syndrome") AND TITLE-ABS-KEY(Estrogen* OR oestrogen* OR Estradiol OR Estriol OR promestriene) AND PUBYEAR > 2000 AND PUBYEAR < 2025 AND ( LIMIT-TO ( LANGUAGE , "English" ) )
Web of Science n = 446 results	TS=("Breast Neoplasm*" OR "Breast Tumor*" OR "Breast Cancer*" OR "Breast Carcinoma*" OR "Cancer of Breast" OR "Cancer of the Breast" OR "Malignant Neoplasm of Breast" OR "Breast Malignant Neoplasm*" OR "Malignant Tumor of Breast" OR "Breast Malignant Tumor*" OR "Mammary Cancer*" OR "Mammary Neoplasm*" OR "Mammary Carcinoma*") AND TS=((Atroph* AND (Vagin* OR Vulva* OR urogenital OR vulvovagin*)) OR "genitourinary syndrome") AND TS=(Estrogen* OR oestrogen* OR Estradiol OR Estriol OR promestriene) AND PY=(2001 OR 2002 OR 2003 OR 2004 OR 2005 OR 2006 OR 2007 OR 2008 OR 2009 OR 2010 OR 2011 OR 2012 OR 2013 OR 2014 OR 2015 OR 2016 OR 2017 OR 2018 OR 2019 OR 2020 OR 2021 OR 2022 OR 2023 OR 2024)

**Table 2S:** Bias risk assessment of included studies accordingly to Newcastle-Ottawa Scale (NOS)

Autor, year and country	Type of study	Selection	Comparability	Outcome	Total
O’Meara et al. (2001)^(8)^ USA	Cohort	4	2	2	8
Durna et al. (2002)^(9)^ Australia	Cohort	2	2	3	7
Dew et al. (2003)^(10)^ Australia	Cohort	3	1	3	7
Le Ray et al. (2012)^(11)^ United Kingdom	Case-control	2	1	1	4
Cold et al. (2022)^(12)^ Denmark	Cohort	4	2	2	8
Agrawal et al. (2023)^(13)^ USA	Cohort	4	2	2	8
McVicker et al. (2024)^(14)^ Scotland and Wales	Cohort	4	2	2	8
